# A checklist of spiders from Yongxing Island, South China Sea, with taxonomic notes on four species of goblin spiders

**DOI:** 10.3897/BDJ.9.e67087

**Published:** 2021-05-21

**Authors:** Jiaxin Tang, Wei Liang, Haitao Shi, Caixia Gao, Shuqiang Li, Guo Zheng

**Affiliations:** 1 College of Life Science, Shenyang Normal University, Shenyang, China College of Life Science, Shenyang Normal University Shenyang China; 2 Ministry of Education Key Laboratory for Ecology of Tropical Islands, Hainan Normal University, Haikou, China Ministry of Education Key Laboratory for Ecology of Tropical Islands, Hainan Normal University Haikou China; 3 Institute of Zoology, Chinese Academy of Sciences, Beijing, China Institute of Zoology, Chinese Academy of Sciences Beijing China

**Keywords:** Araneae, checklist, Oonopidae, new records, Yongxing Islands

## Abstract

**Background:**

Yongxing Island (about 1.85 km^2^) is**the largest island of the Xisha Islands. It is located in the Western South China Sea and belongs to the tropical ocean monsoon climate zone. Yongxing Island is quite rich in biological resources, for example, plants and birds which have been well documented. However, there are limited reports on spider resources in Yongxing Island.

**New information:**

A preliminary checklist of spiders of the Yongxing Island is provided, based on a short-term study undertaken in January 2008. A total of 23 species, belonging to 21 genera and 11 families, were recorded from the area, which forms baseline information of spiders of the Yongxing Island. Amongst these, Oonopidae, Pholcidae, Araneidae and Salticidae were found to have more species in the area. *Brignolia parumpunctata* (Simon, 1893), *Opopaea apicalis* (Simon, 1893), *Opopaea deserticola* Simon, 1891 and *Xyphinus baehrae* Kranz-Baltensperger, 2014 were firstly reported from China, for which we provide taxonomic description in this paper.

## Introduction

**Figure 1.**
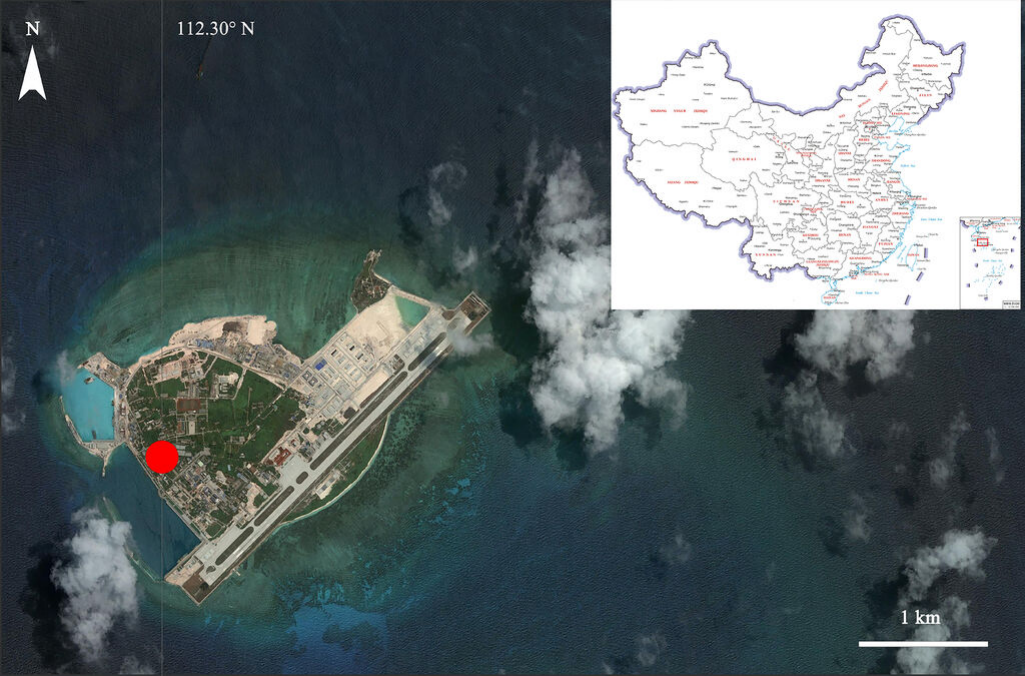
Map showing the survey site in Yongxing Island, South China Sea.

Yongxing Island (Fig. 1), also known as Woody Island of the Paracel Islands and originating from coral reef, is**the largest island of the Xisha Islands (Ye 1996). This area belongs to tropical marine monsoon climate, characterised by dampness and heat (Zhao et al. 1994). The annual mean lowest and highest temperatures are 22.9°C and 28.9°C and the annual rainfall is about 1505 mm (Zhao et al. 2017). Soil of Yongxing Island consists of phosphatic lime type and was formed in the late Holocene (Yu et al. 1995). The above environmental conditions endow the Island with suitable natural habitat and high biodiversity potential. In the sea, tropical marine organisms abound, while on land, tropical plants are luxuriant, with many bird species and other terrestrial organisms (Zhao et al. 1994). Nevertheless, the reports of spiders in the locality are extremely limited up to now.

Oonopids are tiny haplogyne spiders with usually six eyes often gathered together (Tong 2013). According to World Spider Catalog (2021), the family consists of 1874 species in 114 genera distributed mainly in the Tropics and Subtropical Regions. Up to now, a total of 14 genera with 85 species of oonopids are known in China (World Spider Catalog 2021). In the present paper, four species belonging to three genera are reported and illustrated from China for the first time, thereby increasing the total of oonopids to 89 species.

## Materials and methods

Specimens were collected through intensive hand searching and afterwards stored in 75% alcohol and examined using a Leica M205C stereomicroscope. Further details were studied under a Leica DM2500 compound microscope. All illustrations were made using a drawing tube and inked on ink jet plotter paper. Vulvae of females were cleared in lactic acid.

The following abbreviations are used in the text: ALE-anterior lateral eyes; PLE-posterior lateral eyes; IZCAS-Institute of Zoology, Chinese Academy of Sciences in Beijing.

## Taxon treatments

### Brignolia parumpunctata

(Simon, 1893)

01306AB3-2B36-58A4-B607-649B2DA8D24F

#### Materials

- **Type status:** Other material. **Occurrence:** recordedBy: Shuqiang Li; individualCount: 2; sex: 1 male, 1 female; lifeStage: adult; **Taxon:** scientificName: *Brignolia parumpunctata* (Simon, 1893); kingdom: Animalia; phylum: Arthropoda; class: Arachnida; order: Araneae; family: Oonopidae; genus: *Brignolia*; taxonomicStatus: accepted; **Location:** country: China; countryCode: CHN; stateProvince: Hainan; county: Yongxing; decimalLatitude: 16.833; decimalLongitude: 112.333; **Identification:** identifiedBy: Jiaxin Tang; identificationReferences: Platnick et al., 2011 & Ranasinghe & Benjamin, 2016; **Event:** year: 2008; month: 1; day: 13–19

#### Description

**Male.** Measurements (in mm): Body length 1.28; carapace 0.70 length, 0.55 width; abdomen 0.85 length, 0.60 width. Leg measurements: I 2.03 (0.61, 0.29, 0.48, 0.40, 0.25), II 1.76 (0.60, 0.16, 0.55, 0.25, 0.20), III 1.65 (0.55, 0.22, 0.36, 0.34, 0.18), IV 1.69 (0.59, 0.25, 0.34, 0.30, 0.21). Leg formula: 1 > 2 > 4 > 3.

**Figure 2.**
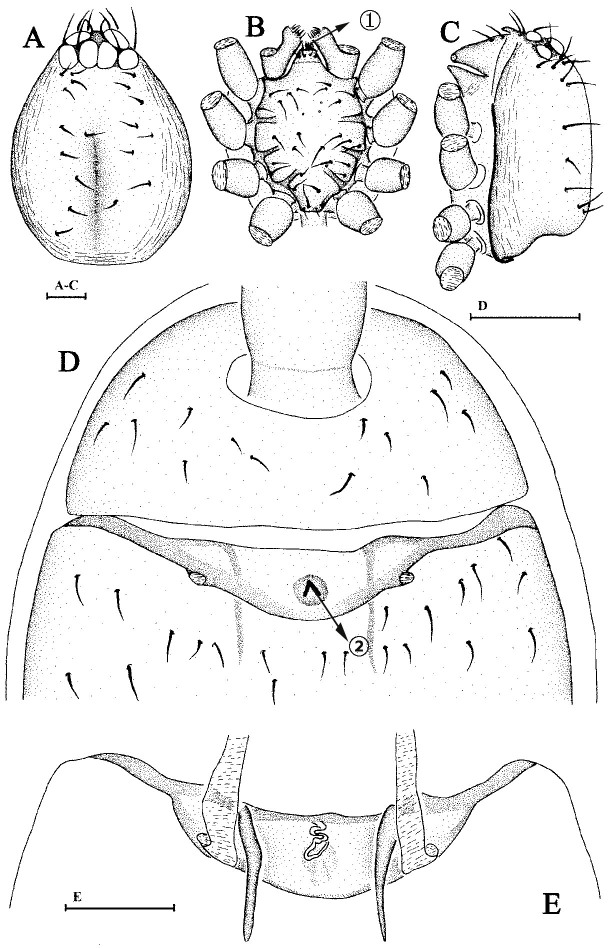
*Brignolia parumpunctata *(Simon, 1893). **A. **Carapace of male, dorsal view; **B.** Same, ventral view; **C.** Same, lateral view, arrow ① points at membranous tips; **D.** Ventral scutum of female, ventral view, arrow ② points at V-shaped ridge; **E.** Genital area of female, dorsal view. Scale bars: 0.1 mm.

Cephalothorax. Carapace and sternum yellow; legs and abdomen pale yellow; chelicerae brownish-yellow. Sides of carapace with finely longitudinal striae; dorsal area smooth with some mesially pointing hairs at lateral edges. Eyes six in two rows, rather large, nearly equally-sized; posterior eyes in a straight row, touching each other (Fig. 2A). Base of fang without ornament (Fig. 2C). Labium wider than long, endites with membranous tips (Fig. 2B).

Legs. Leg with distinct hairs: femur with a row of short ventral setae, 2 prolateral setae; patella without setae; tibia with 2 prolateral setae, 1 retrolateral seta and 1–2 trichobothria; metatarsus with 2 long dorsal setae.

Abdomen. Dorsal scutum oval-shaped, covering nearly whole abdomen. Lobes on anterolateral corners of petiolar tube distinct, ridges developed, but without forming a scutal cove.

**Figure 3.**
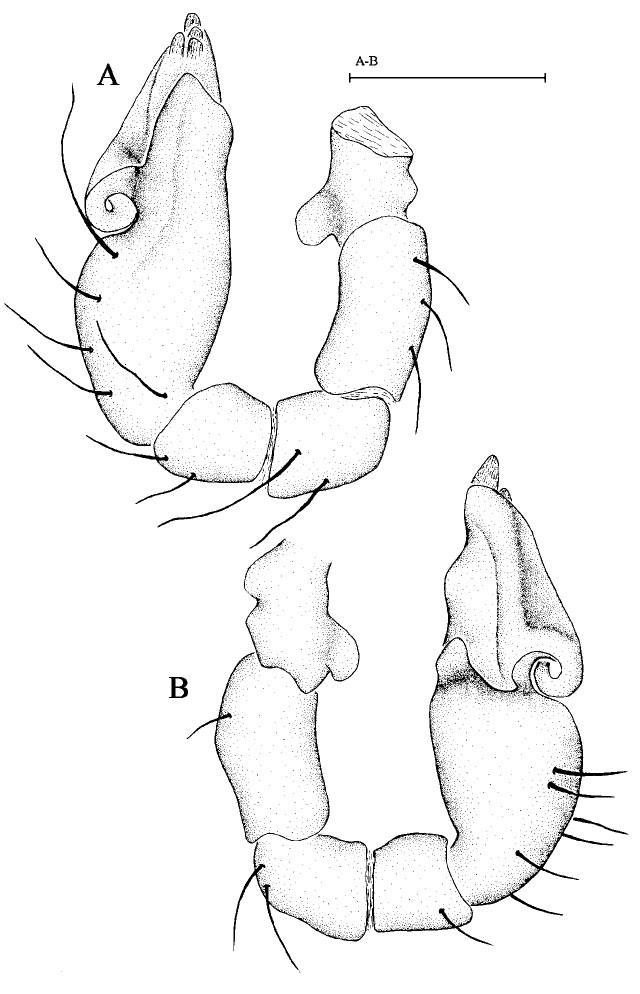
*Brignolia parumpunctata * (Simon, 1893), male. **A.** Left male pale in prolateral view; **B.** Left male palp in retrolateral view. Scale bar: 0.1 mm.

Male palp. Palp (Fig. 3A, B) minute, strongly sclerotised. Palp cymbium dark yellow, bulb dark yellow. Palp trochanter normal, with ventral projection. Palpal patella shorter than femur, not enlarged, unmodified. Palp cymbium narrow in dorsal view, cymbium and bulb incompletely fused, with seam visible in retrolateral view, covered with setae. Bulb of palp elongated, gradually tapering apically, obtusely bent before apex. Embolic part not divided into distinct lobes, bearing some membranous outgrowths.

**Female.**As in male, except as noted. Slightly larger than male. Measurements (in mm): Body length 1.34; carapace 0.65 length, 0.50 width; abdomen 1.00 length, 0.70 width. Leg measurements: I 1.61 (0.55, 0.20, 0.36, 0.28, 0.22), II 1.61 (0.50, 0.26, 0.34, 0.31, 0.20), III 1.43 (0.45, 0.18, 0.33, 0.30, 0.17), IV 1.91 (0.55, 0.28, 0.44, 0.39, 0.25).

Epigynum. Genital area with a small knoblike projection, most of which showing inverted V-shaped ridges (Fig. 2D), a few are inverted Y-shaped. Two internal apodemes rise from the anterior border of post epigastric furrow; a strongly-twisted duct runs from the anterior border of the postepigastric furrow ending in the middle of the knob-like projection (Fig. 2E).

#### Distribution

America, Australia, China (new record), Gambia, India, Indonesia, Pakistan, Pacific Is., Philippines, Seychelles, Sierra Leone, Sri Lanka, Yemen.

### Opopaea apicalis

(Simon, 1893)

89E23A08-4001-52BE-9092-4C79D95C263E

#### Materials

- **Type status:** Other material. **Occurrence:** recordedBy: Shuqiang Li; individualCount: 13; sex: 2 males, 11 females; lifeStage: adult; **Taxon:** scientificName: *Opopaea apicalis* (Simon, 1893); kingdom: Animalia; phylum: Arthropoda; class: Arachnida; order: Araneae; family: Onopidae; genus: *Opopaea*; taxonomicStatus: accepted; **Location:** country: China; countryCode: CHN; stateProvince: Hainan; county: Yongxing; decimalLatitude: 16.833; decimalLongitude: 112.333; **Identification:** identifiedBy: Jiaxin Tang; identificationReferences: Platnick & Dupérré, 2009; **Event:** year: 2008; month: 1; day: 13–19

#### Description

**Male.** Measurements (in mm): Body length 1.35; carapace 0.60 length, 0.40 width; abdomen 0.70 length, 0.35 width. Leg measurements: I 2.15 (0.55, 0.25, 0.40, 0.50, 0.45), II 1.70 (0.55, 0.25, 0.35, 0.30, 0.25), III 1.01 (0.40, 0.18, 0.25, 0.18), IV 1.75 (0.50, 0.25, 0.40, 0.35, 0.25). Leg formula: 1 > 4> 2 > 3.

**Figure 4.**
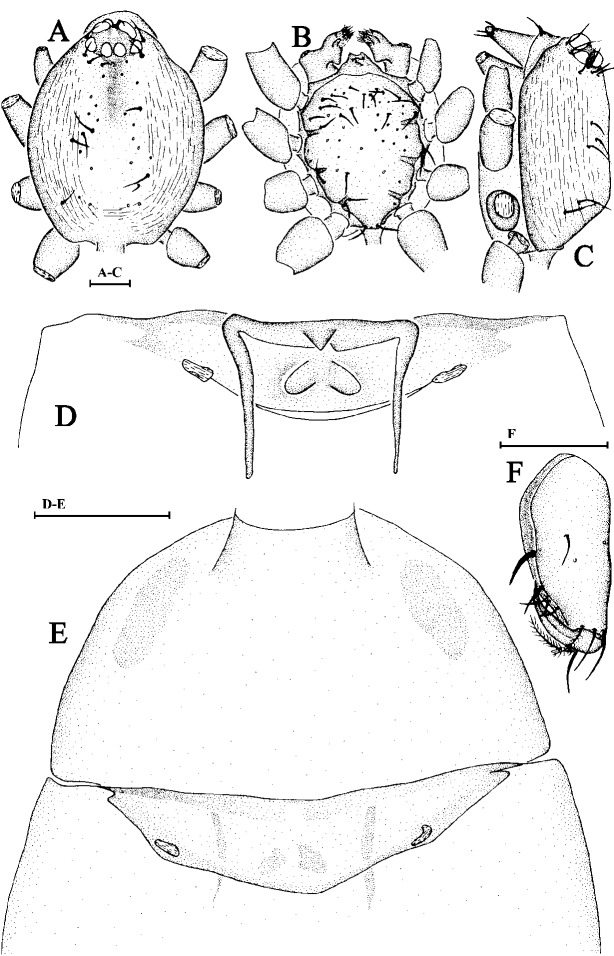
*Opopaea apicalis * (Simon, 1893). **A.** Carapace of male, dorsal view; **B.** Same, ventral view; **C. **Same, lateral view; **D.** Genital area of female, dorsal view; **E.** Ventral scutum of female, ventral view; **F.** Right male chelicerae, posterior view. Scale bars: 0.1 mm.

Cephalothorax. Sides of carapace yellowish-brown; dorsally yellow; chelicerae, sternum yellow; legs and abdomen light yellow. Carapace with a dark brown patch behind eyes, dorsally with a few rows of short hairs. Sides of carapace with finely longitudinal striae. Eyes six in two rows, rather large, nearly equally-sized, ALE slightly separated, touching posterior lateral eyes; posterior eyes in procurved row, touching each other (Fig. 4A and C). Base of fang with two plumose hairs on lateral sides (Fig. 4F). Labium wider than long, endites with membranous tip (Fig. 4B).

Legs. Leg I: femur with a row of dorsal setae, 3 retrolateral setae; patella without setae; tibia with a ventral seta; tarsus with distinct strong setae. Leg II, III and IV similar to leg I.

Abdomen. Dorsal scutum oval-shaped, covering nearly entire abdominal length. Lobes on anterolateral corners of petiolar tube distinct, ridges developed, but without forming a scutal cove; opercula small, oval-shaped. Sperm pore clearly discernible, transverse.

**Figure 5.**
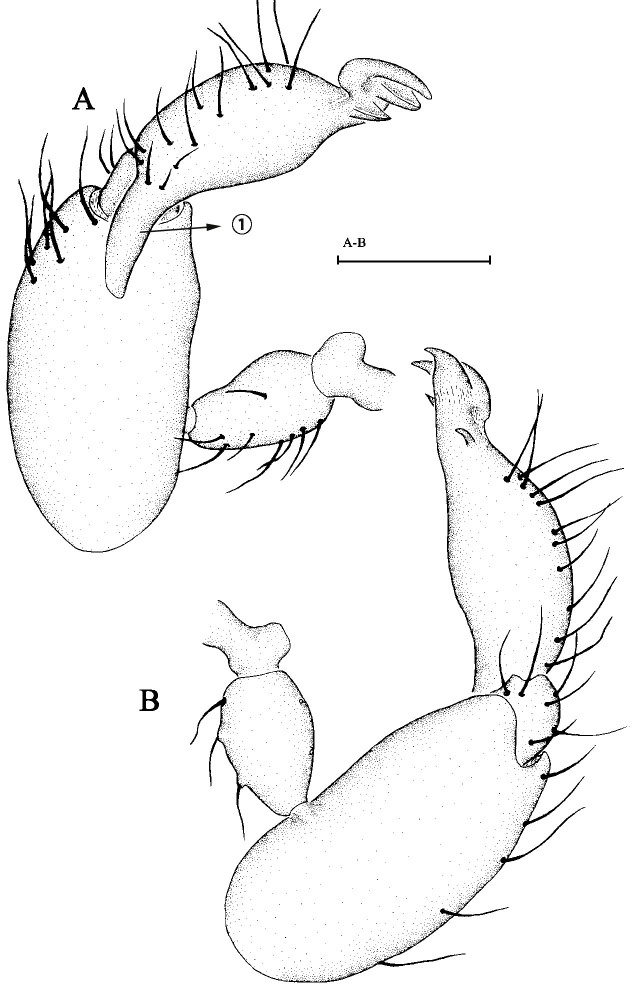
*Opopaea apicalis * (Simon, 1893). **A.** Left male palp in prolateral view, arrow ① points at clavate protrusion; **B.** Left male palp in retrolateral view. Scale bar: 0.1 mm.

Male palp. Patella of palp (Fig. 5A and B) significantly larger than cymbiobulbus; cymbiobulbus with a clavate protrusion at base and three branches at the end.

**Female. **As in male, except as noted. Slightly larger than male. Measurements (in mm): Body length 1.35–1.45; carapace 0.40–0.60 length, 0.35–0.40 width; abdomen 0.50–0.65 length, 0.40–0.45 width. Leg measurements: I 1.88 (0.55, 0.25, 0.40, 0.48, 0.20), II 1.50 (0.50, 0.25, 0.30, 0.25, 0.20), III 1.38 (0.40, 0.20, 0.30, 0.30, 0.18), IV 1.79 (0.54, 0.30, 0.40, 0.35, 0.20).

Epigynum. Postgynal depression of epigastric area shallow, with inverted V-shaped sclerotisation situated posterior to epigastric furrow; parmula black, small (Fig. 4D and E).

#### Distribution

China (new record), Ecuador, Indonesia, Mexico, Pacific Is., Panama, Philippines, Seychelles, Thailand, USA.

### Opopaea deserticola

Simon,891

06A992E2-57F7-554E-B855-72A7EDBD6A13

#### Materials

- **Type status:** Other material. **Occurrence:** recordedBy: Shuqinag Li; individualCount: 3; sex: 1 male, 2 females; lifeStage: adults; **Taxon:** scientificName: *Opopaea deserticola* Simon,1891; kingdom: Animalia; phylum: Arthropoda; class: Arachnida; order: Araneae; family: Oonopidae; genus: *Opopaea*; taxonomicStatus: accepted; **Location:** country: China; countryCode: CHN; stateProvince: Hainan; county: Yongxing; decimalLatitude: 16.833; decimalLongitude: 112.333; **Identification:** identifiedBy: Jiaixn Tang; identificationReferences: Saaristo, 2001; **Event:** year: 2008; month: 1; day: 13–19

#### Description

**Male.** Measurements (in mm): Body length 1.45; carapace 0.65 length, 0.60 width; abdomen 0.90 length, 0.70 width. Leg measurements: I 2.14 (0.62, 0.37, 0.54, 0.40, 0.21), II 1.56 (0.54, 0.27, 0.33, 0.25, 0.17), III 1.98 (0.64, 0.30, 0.43, 0.35, 0.26), IV 1.84 (0.58, 0.26, 0.35, 0.40, 0.25). Leg formula: 1> 3 > 4 > 2.

**Figure 6.**
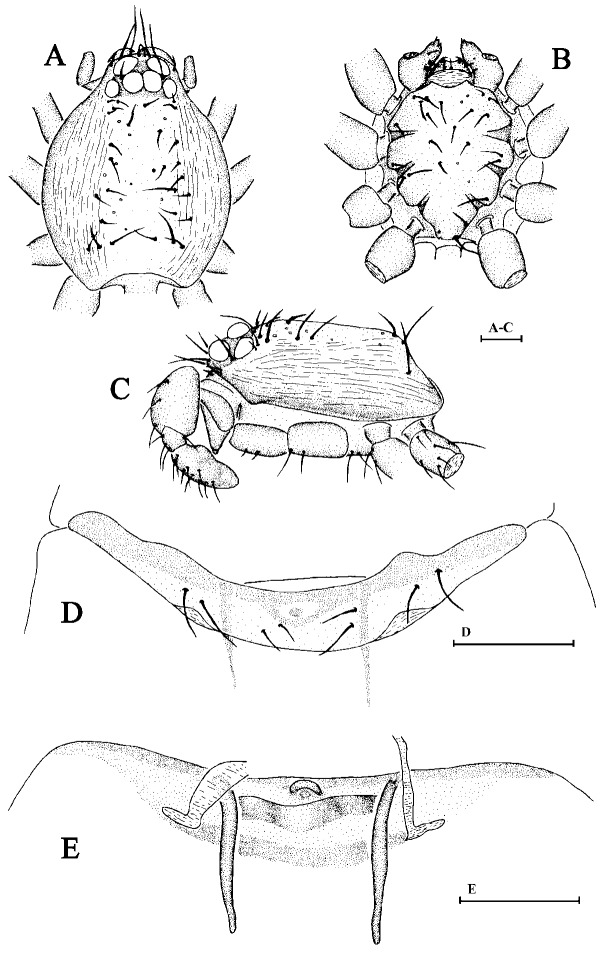
*Opopaea deserticola * Simon, 1893. **A.** Carapace of male, dorsal view; **B.** Same, ventral view; **C.** Same, lateral view; **D.** Ventral scutum of female, ventral view; **E.** Genital area of female, dorsal view. Scale bars: 0.1 mm.

Cephalothorax. Sides of carapace yellowish-brown; dorsally deep yellow; scutum yellow; chelicerae, sternum, legs and ventral scutum yellow. Sides of carapace with finely longitudinal striae; dorsal area smooth with some mesially pointing hairs at lateral edges. Eyes rather large, PLE relatively smaller; compactly arranged, ALE slightly separated, touching posterior lateral eyes; posterior eyes in slightly recurved row, touching each other Fig. 6A and C). Base of fang without ornaments. Labium wider than long, endites with pointed tip (Fig. 6B).

Legs. Leg I: femur smooth, with some hairs; patella with a ventral seta; tibia with distinct hairs, a row of ventral setae and 2–3 trichobothria; tarsus with distinct strong setae. Leg II, III and IV similar to leg I, except femur II with a ventral setae.

Abdomen. Dorsal scutum oval-shaped, covering nearly entirely abdominal length. Lobes on anterolateral corners of petiolar tube distinct, ridges developed, forming a scutal cove.

**Figure 7.**
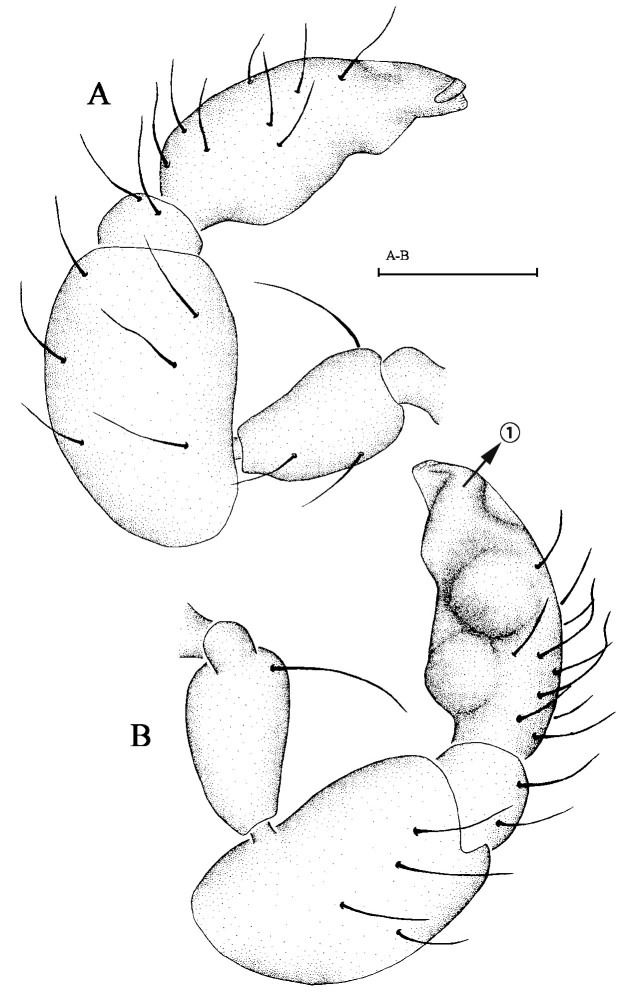
*Opopaea deserticola * Simon, 1893. male. **A** Left male palp in prolateral view; **B.** Left male palp in retrolateral view, arrow ① points at the curving extension. Scale bar: 0.1 mm.

Male palp (Fig. 7A and B). Patella of palp significantly larger than cymbiobulbus; cymbiobulbus with two protrusions at middle and a curving extension to the end.

**Female. **As in male, except as noted. Measurements (in mm): Body length 1.65–1.70; carapace 0.70–0.74 length, 0.60–0.65 width; abdomen 1.00–1.30 length, 0.70–0.80 width. Leg measurements: I 2.00 (0.60, 0.30, 0.45, 0.40, 0.25), II 1.80 (0.60, 0.25, 0.40, 0.35, 0.20), III 1.70 (0.50, 0.25, 0.35, 0.40, 0.20), IV 2.15 (0.65, 0.30, 0.50, 0.45, 0.25).

Epigynum. Postgynal depression of epigastric area shallow; parmula black (Fig. 6D and E).

#### Distribution

Brazil, Caribbean, China (new record), Germany, Japan, Middle East, Pacific Is., Philippines, Spain, USA to Panama, Venezuela.

### Xyphinus baehrae

Kranz-Baltensperger, 2014

C6D35EB9-DB4F-5E52-9330-7D8E0F3F07C7

#### Materials

- **Type status:** Other material. **Occurrence:** recordedBy: Shuqiang Li; individualCount: 4; sex: 1 male, 3 females; lifeStage: adult; **Taxon:** scientificName: *Xyphinus baehrae* Kranz-Baltensperger, 2014; kingdom: Animalia; phylum: Arthropoda; class: Arachnida; order: Araneae; family: Onopidae; genus: *Xyphinus*; taxonomicStatus: accepted; **Location:** country: China; countryCode: CHN; stateProvince: Hainan; county: Yongxing; decimalLatitude: 16.833; decimalLongitude: 112.333; **Identification:** identifiedBy: Jiaixn Tang; identificationReferences: Kranz-Baltensperger, 2014; **Event:** year: 2008; month: 1; day: 13–19

#### Description

**Male.** Measurements (in mm): Body length 1.30; carapace 0.70 length, 0.50 width; abdomen 1.00 length, 0.50 width. Leg measurements: I 2.73 (0.80, 0.40, 0.60, 0.61, 0.32), II 2.55 (0.70, 0.30, 0.60, 0.65, 0.30), III 2.35 (0.70, 0.30, 0.55, 0.55, 0.25), IV 3.17 (0.90, 0.50, 0.70, 0.65, 0.42). Leg formula: 4> 1 > 2 > 3.

**Figure 8.**
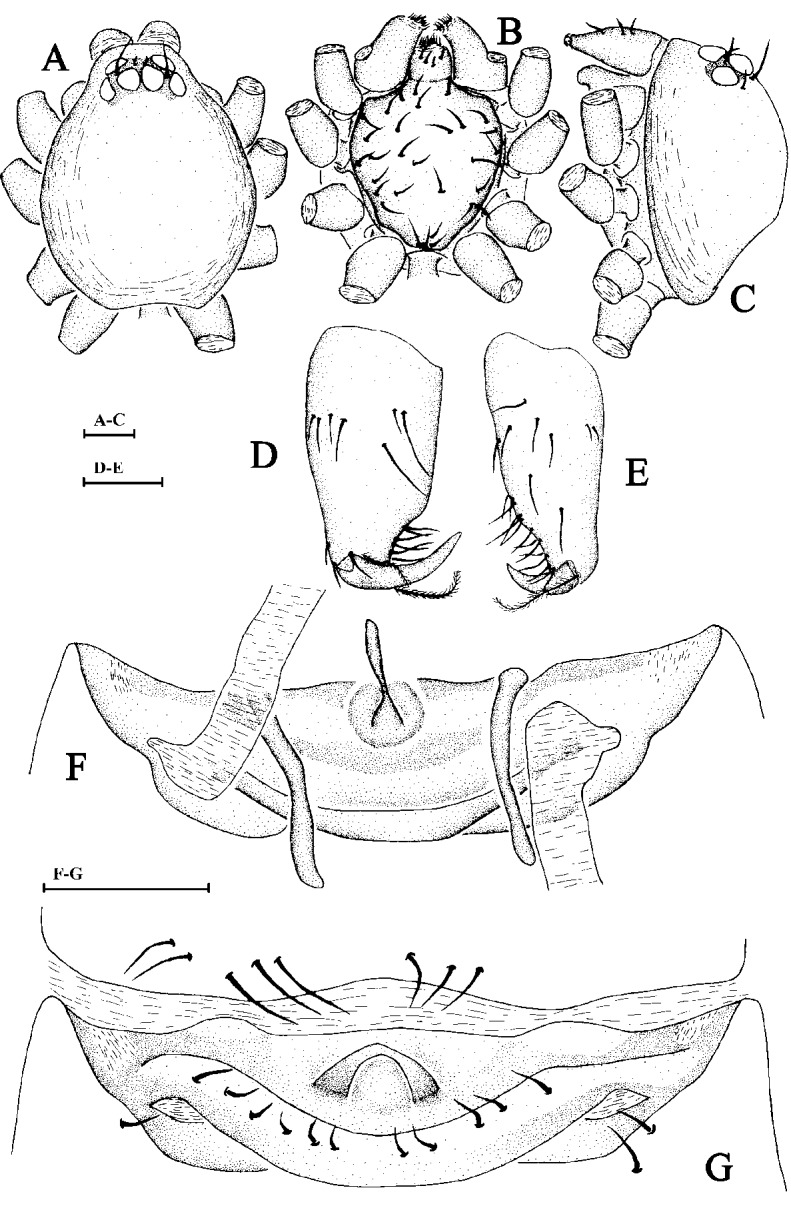
*Xyphinus baehrae * Kranz-Baltensperger, 2014. **A.** Carapace of male, dorsal view; **B.** Same, ventral view; **C.** Same, lateral view; **D.** Left male chelicerae, posterior view; **E.** Left male chelicerae, frontal view; **F.** Genital area of female, dorsal view; **G.** Ventral scutum of female, ventral view. Scale bars: 0.1 mm.

Cephalothorax. Carapace yellow-grey; chelicerae yellow to brownish-yellow; sternum yellow; legs and abdomen light yellow. Sides of carapace with reticulate veins, dorsal area without hairs. Margin of carapace without distinct setae or denticle. Eyes six in two rows, rather large, nearly equally-sized, compactly arranged, ALE slightly separated, posterior eyes in slightly retrocurved row, touching each other (Fig. 8A and, C). Base of fang with two plumose hairs on lateral sides (Fig. 8D and E). Labium wider than long, endites with membranous tip (Fig. 8B).

Legs. Leg I: femur with 2 rows of setae; patella without setae; tibia with 2 ventral setae, 2 dorsal setae and a dorsal trichobothrium; tarsus without setae. Leg II, III and IV similar to leg I.

Abdomen. Dorsal scutum oval-shaped, covering nearly entirely abdominal length. Lobes on anterolateral corners of petiolar tube distinct, ridges developed, but without forming a scutal cove; opercula large, oval-shaped.

**Figure 9.**
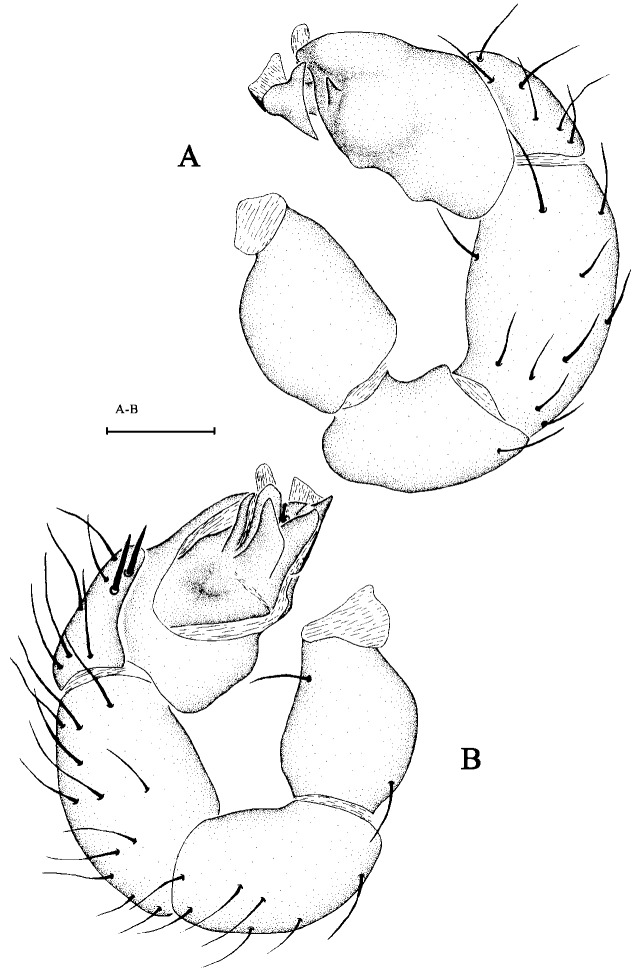
*Xyphinus baehrae * Kranz-Baltensperger, 2014. male. **A.** Left male palp in retrolateral view; **B.** Left male palp in prolateral view. Scale bar: 0.1 mm.

Male palp. Cymbium separated from bulb, with two robust spurs. Bulb with numerous membranous outgrowths on terminal part (Fig. 9A, B).

**Female. **As in male, except as noted. Tibiae with three trichobothria. Measurements (in mm): Body length 1.75–1.90; carapace 0.78–0.90 length, 0.65–0.70 width; abdomen 1.10–1.35 length, 0.45–0.60 width. Leg measurements: I 2.33 (0.60, 0.32, 0.50, 0.48, 0.43), II 4.48 (0.60, 0.30, 0.50, 0.50, 0.25), III 2.01 (0.55, 0.27, 0.50, 0.45, 0.24), IV 2.80 (0.80, 0.31, 0.65, 0.65, 0.39).

Epigynum. Postgynal depression of epigastric area shallow. An arc process visible originating from near the middle of epigastric sulcus (Fig. 8F and G).

#### Distribution

China (new record),**India to Australia.

## Checklists

### Checklist of spiders (Araneae) in Yongxing Island

#### Argiope macrochoera

Thorell, 1891

05DE6ECF-6BE6-56B5-825F-88B030F520B3

##### Materials

- **Type status:** Other material. **Occurrence:** individualCount: 2; sex: female; **Taxon:** family: Araneidae

##### Diagnosis

see Levi (1983)

#### Gasteracantha hasselti

C. L. Koch, 1837

497000D3-7B38-500A-83A9-3D39BAB98542

##### Materials

- **Type status:** Other material. **Occurrence:** individualCount: 2; sex: 1 male, 1 female; **Taxon:** family: Araneidae

##### Diagnosis

see Williams (2017)

#### Thelacantha brevispina

(Doleschall, 1857)

969D935E-82B9-5A35-9B23-1F8999B4D513

##### Materials

- **Type status:** Other material. **Occurrence:** individualCount: 2; sex: 1 male, 1 female; **Taxon:** family: Araneidae

##### Diagnosis

see Emerit (1974)

#### Marinarozelotes jaxartensis

(Kroneberg, 1875)

9E461BA9-F17E-5DCD-9A94-7C501CD9998A

##### Materials

- **Type status:** Other material. **Occurrence:** individualCount: 2; sex: 1 male, 1 female; **Taxon:** family: Gnaphosidae

##### Diagnosis

see Ponomarev and Shmatko (2020)

#### Wadicosa fidelis

(O. Pickard-Cambridge, 1872)

CFF3C302-2FF2-57A6-8964-6DF107D057FC

##### Materials

- **Type status:** Other material. **Occurrence:** individualCount: 1; sex: male; **Taxon:** family: Lycosidae

##### Diagnosis

see Kronestedt and Zyuzin (2009)

#### Brignolia parumpunctata

(Simon, 1893)

46794BEF-6CE8-564E-B93E-02AA5B18CC98

##### Materials

- **Type status:** Other material. **Occurrence:** individualCount: 2; sex: 1 male, 1 female; **Taxon:** family: Onopidae

##### Diagnosis

see Platnick et al. (2011), Ranasinghe and Benjamin (2016)

#### Opopaea apicalis

(Simon, 1893)

9E4911DC-C9E4-5C2E-9479-CBEDAA697B53

##### Materials

- **Type status:** Other material. **Occurrence:** individualCount: 13; sex: 2 males, 11 females; **Taxon:** family: Onopidae

##### Diagnosis

see Platnick and Dupérré (2009)

#### Opopaea deserticola

Simon, 1891

3DBC124B-1AC9-57EC-B387-16ECF6989652

##### Materials

- **Type status:** Other material. **Occurrence:** individualCount: 3; sex: 1 male, 2 females; **Taxon:** family: Onopidae

##### Diagnosis

see Saaristo (2001)

#### Xyphinus baehrae

Kranz-Baltensperger, 2014

09040ADF-81A7-5ED4-9C9B-9F67C721C17B

##### Materials

- **Type status:** Other material. **Occurrence:** individualCount: 2; sex: 1 male, 1 female; **Taxon:** family: Onopidae

##### Diagnosis

see Kranz-Baltensperger (2014)

#### Oxyopes javanus

Thorell, 1887

5E359C47-A831-522B-BB73-2168680772D8

##### Materials

- **Type status:** Other material. **Occurrence:** individualCount: 2; sex: 1 male, 1 female; **Taxon:** family: Oxyopidae

##### Diagnosis

see Sherriffs (1951)

#### Artema atlanta

Walckenaer, 1837

6B4D5570-944A-5706-B31A-1A93F6EF6E2C

##### Materials

- **Type status:** Other material. **Occurrence:** individualCount: 2; sex: 1 male, 1 female; **Taxon:** family: Pholcidae

##### Diagnosis

see Gao and Li (2010)

#### Pholcus manueli

Gertsch, 1937

97BE3AAD-0C1A-5946-B86D-48EF0C0DFFA7

##### Materials

- **Type status:** Other material. **Occurrence:** individualCount: 2; sex: 1 male, 1 female; **Taxon:** family: Pholcidae

##### Diagnosis

see Zhang and Zhu (2009)

#### Pholcus suizhongicus

Zhu and Song, 1999

95E583BC-335C-543B-B2DF-347A882AC5F4

##### Materials

- **Type status:** Other material. **Occurrence:** individualCount: 2; sex: 1 male, 1 female; **Taxon:** family: Pholcidae

##### Diagnosis

see Yao and Li (2012)

#### Smeringopus pallidus

(Blackwall, 1858)

1A4DA0EE-5961-57A4-9B63-EC3063D3D27E

##### Materials

- **Type status:** Other material. **Occurrence:** individualCount: 2; sex: 1 male, 1 female; **Taxon:** family: Pholcidae

##### Diagnosis

see Saaristo (1978)

#### Hasarius adansoni

(Audouin, 1826)

77A251BF-CA91-5922-ABFE-299A98A6EFD2

##### Materials

- **Type status:** Other material. **Occurrence:** individualCount: 2; sex: 1 male, 1 female; **Taxon:** family: Salticidae

##### Diagnosis

see Yin and Wang (1979)

#### Plexippus paykulli

(Audouin, 1826)

D6725B3D-7CB7-552D-8AFD-8CABCF27B3D9

##### Materials

- **Type status:** Other material. **Occurrence:** individualCount: 1; sex: female; **Taxon:** family: Salticidae

##### Diagnosis

see Prószyński (2017)

#### Menemerus bivittatus

(Dufour, 1831)

C26D2CA7-876B-5FEB-8CB1-8934A1211022

##### Materials

- **Type status:** Other material. **Occurrence:** individualCount: 2; sex: 1 male, 1 female; **Taxon:** family: Salticidae

##### Diagnosis

see Żabka (1985)

#### Dictis striatipes

L. Koch, 1872

02711708-40F4-51BC-9770-6A8F37F7D973

##### Materials

- **Type status:** Other material. **Occurrence:** individualCount: 2; sex: 1 male, 1 female; **Taxon:** family: Scytodidae

##### Diagnosis

see Kim and Lee (2018)

#### Argyrodes argentatus

O. P.-Cambridge, 1880

B0A4F45E-3DA5-5B64-8267-ADC68E5803C9

##### Materials

- **Type status:** Other material. **Occurrence:** individualCount: 2; sex: 1 male, 1 female; **Taxon:** family: Theridiidae

##### Diagnosis

see Zhu and Song (1991)

#### Meotipa pulcherrima

(Mello-Leitão, 1917)

E1AD7AB8-6FF4-57A5-9A7F-D278DFF633DE

##### Materials

- **Type status:** Other material. **Occurrence:** individualCount: 2; sex: 1 male, 1 female; **Taxon:** family: Theridiidae

##### Diagnosis

see Yoshida (1993)

#### Coleosoma blandum

O. P.-Cambridge, 1882

9E3388AE-9119-52D3-AAC4-8E8D9D8457A3

##### Materials

- **Type status:** Other material. **Occurrence:** individualCount: 2; sex: 1 male, 1 female; **Taxon:** family: Theridiidae

##### Diagnosis

see Amalin and Barrion (1990)

#### Philoponella prominens

(Bösenberg and Strand, 1906)

065FB1B7-3AFC-5A76-9E03-E4432CF1527D

##### Materials

- **Type status:** Other material. **Occurrence:** individualCount: 2; sex: female; **Taxon:** family: Uloboridae

##### Diagnosis

see Kim and Lee (2013)

#### Tropizodium serraferum

(Lin & Li, 2009)

A3A33973-C548-55E1-AAD7-CCC879637696

##### Materials

- **Type status:** Other material. **Occurrence:** individualCount: 2; sex: 1 male, 1 female; **Taxon:** family: Zodariidae

##### Diagnosis

see Lin and Li (2009)

## Supplementary Material

XML Treatment for Brignolia parumpunctata

XML Treatment for Opopaea apicalis

XML Treatment for Opopaea deserticola

XML Treatment for Xyphinus baehrae

XML Treatment for Argiope macrochoera

XML Treatment for Gasteracantha hasselti

XML Treatment for Thelacantha brevispina

XML Treatment for Marinarozelotes jaxartensis

XML Treatment for Wadicosa fidelis

XML Treatment for Brignolia parumpunctata

XML Treatment for Opopaea apicalis

XML Treatment for Opopaea deserticola

XML Treatment for Xyphinus baehrae

XML Treatment for Oxyopes javanus

XML Treatment for Artema atlanta

XML Treatment for Pholcus manueli

XML Treatment for Pholcus suizhongicus

XML Treatment for Smeringopus pallidus

XML Treatment for Hasarius adansoni

XML Treatment for Plexippus paykulli

XML Treatment for Menemerus bivittatus

XML Treatment for Dictis striatipes

XML Treatment for Argyrodes argentatus

XML Treatment for Meotipa pulcherrima

XML Treatment for Coleosoma blandum

XML Treatment for Philoponella prominens

XML Treatment for Tropizodium serraferum
